# Re-thinking performance assessment for primary care: Opinion of the expert panel on effective ways of investing in health

**DOI:** 10.1080/13814788.2018.1546284

**Published:** 2018-12-12

**Authors:** Dionne Kringos, Sabina Nuti, Christian Anastasy, Margaret Barry, Liubove Murauskiene, Luigi Siciliani, Jan De Maeseneer

**Affiliations:** a Amsterdam Public Health research institute, Department of Public Health, University of Amsterdam, Amsterdam UMC, Amsterdam, The Netherlands;; b Laboratorio Management e Sanità, Institute of Management, Sant'Anna School of Advanced Studies, Pisa, Italy;; c Inspection Générale des Affaires Sociales, Paris, France;; d Health Promotion Research Centre, National University of Ireland Galway, Galway, Ireland;; e Public Health Institute, Vilnius University, Vilnius, Lithuania;; f Department of Economics and Related Studies, University of York, York, UK;; g Department of Family Medicine and Primary Health Care, Ghent University, Ghent, Belgium

**Keywords:** Primary care, health systems, performance assessment, indicators, health information

## Abstract

**Background:** In 2017, the European Commission (EC) identified as a policy priority the performance assessment of primary care systems, which relates to a country’s primary care structure, services delivery and outcomes. The EC requested its Expert Panel on Effective Ways of Investing in Health (Expert Panel) to provide an opinion on ways for improving performance assessment of primary care.

**Objectives:** To provide an overview of domains and dimensions to be taken into consideration in assessing primary care and specific indicators to be collected and analysed to improve understanding of primary care performance.

**Methods:** A sub-group of the Expert Panel performed a literature review. The opinion was drafted, improved and approved through working-group discussions, consultations with the EC, the Expert Group on Health Systems Performance Assessment, and a public hearing.

**Results:** Drawing on the main characteristics of primary care, we propose essential elements of a primary care performance assessment system based on specific indicators. We identified ten domains with accompanying dimensions for which comparative key indicators and descriptive indicators are proposed: (1) universal and accessible care, (2) integrated, (3) person-centred, (4) comprehensive and community-oriented care, (5) provided by a team accountable for addressing a vast majority of personal health needs, (6) sustained partnership with patients and informal caregivers, (7) coordination, (8) continuity of care, (9) primary care organization, and (10) human resources.

**Conclusion:** The identified characteristics and criteria for development of a primary care performance assessment system provides a starting point for strengthening the coherence of assessment frameworks across countries and exchanging best practices.

Key messages

- Performance assessments of primary care systems should cover different core primary care domains measured by comparative and descriptive indicators.
- Indicators need to be aligned with health system objectives and allowed for the routine collection of valid and reliable data to be relevant for a health system.

## Introduction

Primary care can contribute to strengthening the overall health system’s performance by providing affordable, accessible and coordinated care; and by reducing avoidable hospital admissions [1]. This was recently re-emphasized in the State of Health in the EU 2017 Companion Report [2]. Well structured, organized and delivered primary care can play a fundamental role in improving not only population health but also population well-being since it covers both biomedical health needs and the broader contextual or social determinants of health such as social conditions, employment and environment. As such, primary care can be an effective tool to reduce inequities in societies [3–5].

Measuring performance is a prerequisite for monitoring the achievement of a health system and serves to identify opportunities for improvement. Given the complexity of primary care systems (covering its structures, services delivery processes, and outcomes), measuring its performance at national, regional or local level is challenging. Policymakers need tools and methodologies to assess the performance of primary care, to understand how primary care operates and contributes to their health system benefitting all actors in primary care. Primary care providers, including general practitioners/family physicians, require information about relevance, acceptability, quality, person-centredness, effectiveness, and sustainability of their daily care for patients. Almost all countries in Europe carry out recurrent assessments on the performance of primary care in general, or on important parts of the primary care system. However, a wide variety of indicators are used across member countries to measure performance in primary care, and primary care providers are not always part of the developmental phase. It is very important that primary care providers are part of the development of indicator sets, to increase the validity of the performance data, and its potential to contribute to quality improvement initiatives at services delivery level. In many cases, the set of indicators available to policymakers are insufficient or focused on a subset of dimensions, as shown by a recent survey among 21 EU countries [6]. Most countries reported collecting descriptive information about providers, access, and patient-centredness when assessing the performance of primary care. Only half of the countries reported measuring clinical performance. Aspects such as inequalities, workload and primary care workforce satisfaction are even less frequently reported. It could, therefore, be helpful at European level if guidance could be provided to countries on tools and methodologies for adequately assessing the performance of primary care at country level. For general practitioners (GPs), these tools and methodologies could provide a welcome help in its search of professional and scientific organization for useful quality indicators. This would also increase opportunities for international comparisons, and more importantly improve the contribution of primary care to health system outcomes in each country.

In 2017, the European Commission requested its Expert Panel on Effective Ways of Investing in Health (Expert Panel), comprising 14 experts from across the EU, to provide its views on **(**a) domains and dimensions to be taken into consideration in assessing primary care; and **(**b) specific indicators to be collected and analysed to give a better understanding of the performance of primary care [7]. In this study, we report the key messages from the Expert Panel’s opinion on ways of assessing performance in primary care [8].

## Methods

A subgroup of the Expert Panel (authors) identified tools and methodologies based on the scientific literature, classical health system performance approaches applied by international organizations (e.g. OECD, WHO), and existing primary care performance frameworks (e.g. [5,9]). Draft versions of the opinion were regularly discussed with the responsible technical officers of the European Commission’s Directorate General on Health and Food Safety (DG SANTE), representatives of the Expert Group on Health Systems Performance Assessment, and with the broader Expert Panel to build on their professional experiences in primary care policy, practice and research. The resulting opinion was discussed in a public hearing and improved accordingly.

## Results

### A comprehensive definition of primary care captured by ten core domains

Drawing on the existing literature, in 2014 the Expert Panel defined the role and goals of primary care as ‘[…] the provision of universally-accessible, integrated, person-centred, comprehensive health and community services provided by a team of professionals accountable for addressing a large majority of personal health needs. The services are delivered in a sustained partnership with patients and informal caregivers, in the context of family and community, and play a central role in the overall coordination and continuity of people’s care […]’ [10].

From this definition, eight domains of primary care can be identified, i.e. (1) universal and accessible, (2) integrated, (3) person-centred, (4) comprehensive and community-oriented, (5) services provided by a team of professionals accountable for addressing a large majority of personal health needs (quality), (6) sustained partnership with patients and informal caregivers, in the context of family and community, (7) coordination and (8) continuity of people’s care. Additionally, the domains of (9) primary care organization and (10) human resources need to be added since they are key determinants of the delivery of high quality, efficient and equitable primary care services. Table 1 displays some of the key dimensions of each domain. General practitioners will especially recognize that the central aspects of their professional activities: being responsive for bio-psychosocial needs of patients, building relationships and continuity with patients, coordination of chronic care, and making sure that no one is left behind, are well represented in Table 1.

**Table 1.** Primary care domains and dimensions classified by structure, process and outcome.

Primary care domains	Exemplary dimensions
*Structure of primary care*	
Universal and accessible	• Population covered by primary care services • Affordability of primary care services • Geographic availability of primary care services • First-contact accessibility • Accommodation • Timeliness and responsiveness of primary care services (e.g. primary care consultations)
Organization of professionals and workforce	• Needs, supply, profile and planning of primary care workforce • Status and responsibilities of primary care disciplines; role of academic institutions and professional associations • Training and multidisciplinary skill mix • Human resources management, including provider well-being, competence and motivation • Role of nurses and other primary care health professionals (task delegation, substitution, competency sharing) • Role of community pharmacists in PHC and pharmaceutical care • Role and function of managers • Income of primary care workforce • Development of undergraduate and postgraduate specific (interprofessional) training
Primary care organization	• Accountability: a formal link between a group of providers and a defined population (list system, geographical area) • Primary care payment and remuneration system (e.g. capitation, FFS, P4P) • The presence and strength of market forces in primary care • Office and facility infrastructure (e.g. information systems and medical technology, point-of-care testing) • Composition of the interprofessional team • Organizational components of coordination and integration: structure and dynamics (job descriptions and team functioning, management and practice governance, clinical information management, organizational adaptivity and culture (traditional command-and-control versus complex adaptive systems approach), team-based organization • Volume and duration of primary care provider consultations, home visits, and telephone consultations • Organizational aspects of referrals to medical specialists; referrals to specialized trajectories (e.g. in mental health, occupational health) • Quality of management • Primary care budget in relation to total healthcare budget
*Processes of primary care*	
**Primary care domains**	**Exemplary dimensions**
Integrated	• Integration of public health services and approach in primary care: e.g. community-oriented primary care • Integration of pharmaceutical care in primary care • Integration of mental health in primary care • Integration between primary care and social care
Comprehensive and community oriented	• Comprehensiveness of services provided (e.g. health promotion, disease prevention, acute care, reproductive, mother and child healthcare, childhood illness, Infectious illness, chronic care, mental health, palliative care) • Primary care takes into account population and community characteristics • Primary care is integral part of the local community
Sustained partnership with patients and informal caregivers	• Policies for coordination between professionals and informal caregivers • Policies to support informal caregivers • Strategies for patient engagement in care planning over time • Participation of informal care givers/citizens in the development of primary care services • Participatory power of patients/informal care givers/citizens
Coordination of people’s care	• Coordination between primary and secondary care: appropriateness of referrals, gatekeeping, integrated patient records, protocols for patients with chronic conditions • Coordination between primary and social care • Policies for respite care
Continuity of people’s care	• Continuity of care (longitudinal, informational, and relational) • The provision of care throughout the life cycle • Care that continues uninterrupted until resolution of an episode of disease • Role of primary care in continuity and interaction with emergency departments
*Outcomes of primary care*	
**Primary care domains**	**Exemplary dimensions**
Person-centred	• Person-centred care, shared decision-making, focusing on ‘life goals’ of the patient • Patient–provider respect and trust; cultural sensitivity; family centred care • Consider patients/people as key partners in the process of care • Maintain a holistic eco-bio-psychosocial view of individual care
Addressing personal health needs (provide high quality primary care)	• Quality of diagnosis and treatment in primary care for acute and chronic conditions • Quality of care for chronic conditions, maternal and child healthcare • Health promotion; primary and secondary prevention • Patient safety

To manage these domains from a systemic perspective, it has proven useful to apply Donabedian’s general assessment framework of structure-processes-outcomes [11], which applies to every health system and setting (e.g. [8,12]). Each of the ten domains can be classified as a primary care structure, process or outcome, as shown in Table 1. This provides the opportunity to link the primary care setting with the structures, processes and outcomes of other parts of the health system (e.g. hospitals), and thus assess primary care’s overall contribution to health system outcomes.

### Comparative key indicators and additional descriptive indicators

Following the definition of primary care and the identification of the core domains and accompanying dimensions, some indicators can be developed to capture the performance of primary care. The development of these indicators should aim to link primary care providers’ actions to performance results, which in turn allows the monitoring of the achievement of health system outcomes and the identification of future policy developments and improvements.

Indicators can be split into comparative key indicators and descriptive additional indicators. Comparative key indicators are those whose score may be evaluated in comparison with a target or a benchmark (e.g. waiting time for the first visit by a physician). Descriptive (observational) indicators are those whose score provides useful information for decision makers but whose interpretation is potentially ambiguous. For example, the extent to which GPs address mental health problems will not only depend on the comprehensiveness of the approach in the consultation (person-centredness), but also on the prevalence of mental health problems in their patient population (related to e.g. socioeconomic status) and on the fact whether or not there are other primary care providers (e.g. primary care psychologists) available. Nevertheless, this data provides useful information if correctly contextualized in a specific health system and to compare progress within a country over time.

Examples of indicators along the ten domains are provided in Table 2. The comprehensive list of indicators is accessible on the Expert Panel’s website at https://ec.europa.eu/health/expert_panel/home_en [9].

**Table 2.** Examples of comparative key indicators by domains.

Primary care domains	Examples of indicators
*Structure of primary care*	
Universal and accessible	• Total expenditure on primary care as a percentage of total expenditure on health • Amount patients have to pay for a GP/primary care consultation and amount reimbursed • Average number of days waiting to see a GP/primary care provider when confronted with a health problem • Access to pharmacy services 24/7 (number of pharmacies providing on call or night duties)
Human resources in primary care	• Average number of working hours per week of GPs/nurses/pharmacists/social workers • Average age and geographical distribution of practising providers in primary care • Total number of active GPs as a ratio to total number of active physicians • Total number of nurses active in primary care compared to total number of nurses in primary care, secondary and tertiary care
Primary care organization	• Primary care payment system, revenues, and operating costs • Average income of 1FTE GP compared to average income of specialist; of primary care nurse compared to hospital nurse • Clear vision and mission statements of primary care teams • Existence of continuous quality improvement processes e.g. is there a structured periodic communication between local GPs and community pharmacists?
*Processes of primary care*	
**Primary care domains**	**Examples of indicators**
Integrated	• Extent to which GPs/primary care teams carry out health promotion and prevention activities • Extent to which mental health is addressed as part of routine consultations • Is there a structured cooperation between PHC and social care? • To what extent are disciplines like occupational therapy, physiotherapy, speech therapy, integrated in primary care teams?
Comprehensive and community oriented	• Extent to which patients visit a GP for first-contact care for specific health conditions; people with a first convulsion; suicidal inclinations; alcohol addiction problems. • Is FP/GP the only medical discipline in PHC? • Are there activities related to community oriented primary care? • Is palliative care at home organized?
Sustained partnership with patients and informal caregivers	• Per cent of informal caregivers who receive support from primary care • Per cent of patients reporting help by informal caregivers • Presence of organizations of informal caregivers in a community • Mechanisms for patient engagement in healthcare planning and decision-making
Coordination of people’s care	• Is there a gate-keeping system (access to specialists through referral)? • Do patients need a referral to access the paramedical and nursing disciplines, to access social care? • Is it common for GPs to have regular (electronic) face-to-face meetings (e.g. at least once per month) with other healthcare providers (e.g. community mental healthcare workers, medical specialists, etc.) • Is the GP informed about patients’ admission to hospital care on time?
Continuity of people’s care	• Do GP-practices have a patient list system or another form of defined population? • Per cent of patients reporting visiting their usual primary care provider for their common health problems • Per cent of GPs/primary care teams routinely keeping electronic clinical records for all patient contacts. • Do primary care practices receive information within 24 h about contacts that patients have with out-of-hours services?
*Outcomes of primary care*	
**Primary care domains**	**Examples of indicators**
Person-centred	• Duration of regular visit (minutes) of different types of providers • Per cent of patients who rate that they (i) trusted the GP/nurse/social worker/…; (ii) were involved in shared decision-making; (iii) were satisfied with primary care visit • Patient-related experience measures (PREMs) and Patient-related outcome measures (PROMs) collected through a continuous survey to patients • Do patients have access to their electronic health records?
Addressing personal health needs (provide high quality primary care)	• Per cent of infants vaccinated within primary care against e.g. diphtheria; tetanus; per cent of population aged 60+ vaccinated against flu; HPV vaccinations • The defined daily doses of antibiotics use in ambulatory care per 1000 inhabitants • Percentage of diabetic population with blood pressure above 140/90 mmHg observed in the last 12 months • Percentage of patients stating that the treatment contributed to the achievement of their life goals

## Discussion

In this section, we will discuss criteria to guide the selection of indicators, approaches to collect primary care performance measurement data, key aspects of successful development and implementation process of a performance assessment system and possible implications.

### Criteria to guide the selection of indicators

Regardless of who is responsible for the selection of indicators (e.g. policy makers, scientific societies of GPs/family physicians, insurers), the choice of indicators should be guided by, at least, the following criteria: (i) alignment with policy objectives (indicators are to be informative about policy objectives defined by the health system); (ii) ability to routinely collect the information, either from administrative sources or from specifically designed surveys (indicators have more meaning with a time dimension to assess progress); and (iii) validity and reliability of information (indicators need to be based on credible sources and survey instruments need to be validated, for example). For each indicator, each criterion needs to be assessed.

In addition, a performance assessment system will need to cover a ‘reasonable’ number of indicators and targets for primary care. Either an excessive or a low number of performance indicators can result in a weak correlation between performance indicators and performance itself [13]. The confusion generated by many targets might disorient the actors of the organization who may then behave differently from the priority actions. Alternatively, a minimal number of targets may induce tunnel vision as a consequence of narrowing the managerial attention only to some aspects of the global performance [14]. Finally, an appropriate understanding and interpretation of the data often requires additional qualitative data collection e.g. describing patients’ expectations and experiences, apart from the quantitative data, measured through indicators. When comparing outcomes of primary care providers or systems, it will be crucial to include variation in context (e.g., data on characteristics of the population and society, the health system, the social welfare system) [15].

### How to collect performance measurement data

Evidence-based data collection and information provision should be the basis of any performance assessment system. This includes professional evidence (based on clinical practice experiences), contextual evidence (considering characteristics of the population, society, health system) and policy evidence (based on policy priorities and impact) [16].

To minimize registration workload for primary care providers, there is a need to explore the use of existing data sets to collect performance measurement data. Whenever feasible, use of administrative data is collected directly from source databases instead of explicit reporting by institutions as it will speed up the collection process and decrease the possibility of errors. When using existing data sets attention will need to be paid to issues such as regulations and privacy (particularly for the individual patient level data) as well as standardization (especially for the population level data). For instance, the increasing potential of electronic patient data could be examined, including electronic prescribing systems that allow for the analysis of safety and effectiveness issues. Electronic patient records, as used by GPs, certainly when they use appropriate coding systems (e.g. International Classification of Primary Care, second edition) can provide extremely valuable information [17]. Through the adoption of new and user-friendly ICTs, it is also increasingly possible to collect evidence directly from patients, users and citizens through systematic and continuous surveys (e.g. PREMs and PROMs).

### Key aspects of successful development and implementation process

The existence of a performance assessment system, even though technical and scientifically sound, does not guarantee its adoption by policymakers and other stakeholders (e.g. GPs). Also, a performance management system can lead to dysfunctional performances (also called performance paradoxes) such as perverse learning—i.e. when organizations or individuals have learned how measurement works and manipulate their performance results [13].

To limit the occurrence of these paradoxes and support a successful implementation and adoption of performance evaluation systems in health, it will be important that performance assessment is primarily applied as a tool to activate a positive comparison, discussion and learning process among primary care providers based on reputation and not on penalty mechanisms [18]. Transparent disclosure of performance information will likely stimulate data peer review and learning and leverages professional reputation [19,20]. According to Hibbard et al., [21] making performance information public stimulates long-term improvements, provided that performance assessment is appropriately contextualized (e.g. through information on case-mix). It is also vital that there is a feeling of ownership and acceptance of performance assessment outcomes by key stakeholders, such as GPs and other primary care providers, healthcare managers and policymakers. This can be created by involving potential users in the development phase of performance indicators [22,23]. Once indicators are selected, they should be based on recent data and fed back to its users (e.g. policymakers and primary care providers) on time, to preserve its actionability (i.e. relevance to act upon). Timeliness of performance data will allow policymakers to make decisions promptly (e.g. correct poor performance or dysfunctional behaviours), and primary care providers to improve the quality of care.

### Implications

It is not expected that any health system will use all identified indicators but rather will select a set that are most relevant to it, taking account of defined criteria. Of course, a compilation of possible indicators is only a first step, and we fully recognize that much more needs to be done. The challenges in many countries are considerable, with primary care being provided by a different combination of health professionals, using a variety of organizational models, in dispersed locations, and often with poorly developed systems of data collection. Moreover, the performance of the primary care system cannot be seen in isolation, given its dependence on the rest of the health system and other sectors, such as social welfare. Nonetheless, the proposed indicators do offer a means to develop and strengthen the existing dialogue among those responsible for primary care across Europe. The European Commission could encourage the development of coherent performance assessment of primary care through the voluntary cooperation between the EU Member States in the exchange of best practice. It is clear that GPs and their societies/associations have a role to play in this process, particularly in the development, implementation, and data collection phases.

## Conclusion

The identified characteristics and criteria for development of a primary care performance assessment system provides a starting point for strengthening the coherence of assessment frameworks across countries and exchanging best practices.
